# EQUITABLE ACCESS TO LATE-LIFE DEPRESSION CARE: A STEPPED WEDGE RCT WITH COMMUNITY ORGANIZATIONS

**DOI:** 10.1093/geroni/igae098.0459

**Published:** 2024-12-31

**Authors:** Lanvin Andres, Lesley Steinman, Mary Mitchell, Emy Haruo

**Affiliations:** IDIC Filipino Senior & Family Services, Seattle, Washington, United States; UW Health Promotion Research Center, Seattle, Washington, United States; Aging and Disability Services - Seattle/King County Area Agency on Aging, Seattle, Washington, United States; Neighborhood House, Seattle, Washington, United States

## Abstract

For the past several years, our community-academic partnership has been collaborating with community-based social service and mental health organizations in Washington and California on the PEARLS Equity Study. The goal of this five-year study is to improve equitable access to late-life depression care (PEARLS) among older adults experiencing poverty who are linguistically diverse, BIPOC, and/or rural dwelling, recognizing many older adults are multiply marginalized by intersecting identities. In phase one (2020), we conducted formative research (39 interviews) with both current and potential PEARLS adopters that reach priority populations. Using a social marketing framework, our guide explored barriers, benefits and process for PEARLS adoption; organization capacities and needs; PEARLS acceptability and adaptations; and preferred communication channels. We conducted thematic analysis using the rapid framework method to develop strategies, collaborations, and adaptations to integrate depression care in ways that aligned with these contexts. In phase two (2021-2023), we evaluated these new dissemination and implementation strategies and tools in a stepped wedge Randomized Controlled Trial (swRCT) to test improvement in equitable access to mental health care. Our primary and secondary outcomes are new PEARLS adopters and reach of priority populations, respectively. We randomized counties into three waves or steps, choosing a stepped wedge design as more feasible for community-engaged research with all organizations ultimately receiving the intervention. We engaged over 500 organizations and 700 providers in outreach, webinars, community conversations, 1:1 support, and training. We are currently analyzing RCT, process and cost evaluation data to present findings and lessons learned at GSA.

